# Supplementary material for: Resolving the full spectrum of human genome variation using Linked-Reads
Source: Genome Res. 2019 Apr;29(4):635–45. doi: 10.1101/gr.234443.118 (PMC6442396; doi:10.1101/gr.234443.118)
Supplement: Supplemental Material [file supp_gr.234443.118_Supplemental_longranger-2.2.zip › longranger-2.2.tar.bz2!/lr2.2-public/lib/stan/cmdstan/stan/doc/api/html/math_2functions_2log__sum__exp_8hpp_source.html]

Stan: src/stan/math/functions/log\_sum\_exp.hpp Source File


|  |  |
| --- | --- |
|  | Stan  2.5.0  probability, sampling & optimization |


- Main Page
- Related Pages
- Namespaces
- Classes
- Files

- File List
- File Members

All Classes Namespaces Files Functions Variables Typedefs Enumerations Enumerator Friends Macros Pages

- src
- stan
- math
- functions

log\_sum\_exp.hpp

Go to the documentation of this file.

1 #ifndef STAN\_\_MATH\_\_FUNCTIONS\_\_LOG\_SUM\_EXP\_HPP

2 #define STAN\_\_MATH\_\_FUNCTIONS\_\_LOG\_SUM\_EXP\_HPP

3

4 #include <stan/math/functions/log1p\_exp.hpp>

5 #include <vector>

6 #include <boost/math/tools/promotion.hpp>

7 #include <limits>

8

9 namespace stan {

10  namespace math {

11

47  template <typename T1, typename T2>

48  inline typename boost::math::tools::promote\_args<T1,T2>::type

49  log\_sum\_exp(const T2& a, const T1& b) {

50  using std::exp;

51  if (a > b)

52  return a + log1p\_exp(b - a);

53  return b + log1p\_exp(a - b);

54  }

55

68  double log\_sum\_exp(const std::vector<double>& x) {

69  using std::numeric\_limits;

70  using std::log;

71  using std::exp;

72  double max = -numeric\_limits<double>::infinity();

73  for (size\_t ii = 0; ii < x.size(); ii++)

74  if (x[ii] > max)

75  max = x[ii];

76

77  double sum = 0.0;

78  for (size\_t ii = 0; ii < x.size(); ii++)

79  if (x[ii] != -numeric\_limits<double>::infinity())

80  sum += exp(x[ii] - max);

81

82  return max + log(sum);

83  }

84

85

86  }

87 }

88

89 #endif

stan::math::log1p\_exp

boost::math::tools::promote\_args< T >::type log1p\_exp(const T a)

Calculates the log of 1 plus the exponential of the specified value without overflow.

**Definition:** log1p\_exp.hpp:44

log1p\_exp.hpp

stan::math::log\_sum\_exp

boost::math::tools::promote\_args< T1, T2 >::type log\_sum\_exp(const T2 &a, const T1 &b)

Calculates the log sum of exponetials without overflow.

**Definition:** log\_sum\_exp.hpp:49

stan::math::exp

Eigen::Matrix< T, Rows, Cols > exp(const Eigen::Matrix< T, Rows, Cols > &m)

Return the element-wise exponentiation of the matrix or vector.

**Definition:** exp.hpp:18

stan::math::max

double max(const double a, const double b)

**Definition:** max.hpp:7

stan::math::log

Eigen::Matrix< T, Rows, Cols > log(const Eigen::Matrix< T, Rows, Cols > &m)

Return the element-wise logarithm of the matrix or vector.

**Definition:** log.hpp:16

stan::math::sum

double sum(std::vector< double > &x)

**Definition:** sum.hpp:10

stan::agrad::log

fvar< T > log(const fvar< T > &x)

**Definition:** log.hpp:15

stan::agrad::exp

fvar< T > exp(const fvar< T > &x)

**Definition:** exp.hpp:16


---

[ Stan Home Page ]

*© 2011–2014,
Stan Development Team.*
